# Disparities in hospice enrollment timing and end-of-life care intensity across non-cancer diagnoses: a 10-year hospital-based cohort study

**DOI:** 10.1080/07853890.2026.2670058

**Published:** 2026-05-16

**Authors:** Chun-Li Wang, Lung-Chun Lee, Chiann-Yi Hsu, Chia-Yen Lin

**Affiliations:** ^a^ Health Management Center, Taichung Veterans General Hospital, Taichung, Taiwan; ^b^ Department of Family Medicine, Taichung Veterans General Hospital, Taichung, Taiwan; ^c^ Institute of Medicine, Chung Shan Medical University, Taichung, Taiwan; ^d^ Biostatistics Group, Department of Medical Research, Taichung Veterans General Hospital, Taichung, Taiwan; ^e^ Department of Urology, Taichung Veterans General Hospital, Taichung, Taiwan; ^f^ School of Medicine, College of Medicine, National Yang Ming Chiao Tung University, Taipei, Taiwan; ^g^ School of Medicine, Chung Shan Medical University, Taichung, Taiwan

**Keywords:** Non-cancer decedents, hospice care, end-of-life care, place of death, aggressive-care, cross-diagnosis comparison

## Abstract

**Introduction:**

Non-cancer patients with advanced illnesses often experience delayed hospice referral and high-intensity care near the end of life, yet cross-diagnosis comparisons remain limited. This study examined diagnosis-specific patterns in hospice use and care intensity among non-cancer decedents.

**Materials and Methods:**

We conducted a 10-year retrospective cohort study of adults with hospice-eligible non-cancer diagnoses who died or were discharged in a moribund condition between 2010 and 2019 at a tertiary referral hospital in Taiwan. Primary outcomes were place of death and an aggressive-care score (0–5) based on five EOL indicators in the last 28 days: hospitalisation >14 days, ≥1 intensive care unit (ICU) admission, ≥1 emergency department (ED) visit, cardiopulmonary resuscitation (CPR), and intubation with mechanical ventilation. Multivariable logistic regression identified factors associated with high aggressive-care scores (≥4).

**Results:**

Among 5,127 non-cancer decedents, 7% received hospice care, with rates varying across diagnostic groups. Most hospice enrolments (60.2%) occurred within 7 days before death. Hospice recipients had lower median aggressive-care scores (2 vs. 3, *p* < 0.001) and lower ICU admissions (39.7% vs. 63.7%), ED visits (62.2% vs. 75.1%), CPR (1.7% vs. 10.1%), and mechanical ventilation (9.2% vs. 23.2%). Advanced heart disease (aOR 1.95) and end-stage renal disease (aOR 1.96) were associated with high aggressive-care scores, while hospice enrolment was associated with lower odds of such scores (aOR 0.46).

**Conclusion:**

Hospice care among hospitalised non-cancer decedents was infrequent and often initiated in the final week across diagnoses. These findings highlight diagnostic differences in end-of-life care intensity and support referral strategies for earlier palliative care integration.

## Key messages

1. This study is among the first to compare aggressive-care scores across multiple non-cancer diagnoses in hospitalised decedents.
2. Using a composite aggressive-care score provides a practical measure for benchmarking end-of-life care intensity in non-cancer populations.
3. Cross-diagnosis assessment reveals disparities that can inform policies to promote equitable hospice access and timely integration of palliative care.

## Introduction

With global population ageing and the rising prevalence of chronic, progressive illnesses, healthcare systems face increasing demand for care that supports quality of life in the final stages of illness [1,2]. High-quality end-of-life (EOL) care is now widely regarded as a core indicator of healthcare system performance, with the World Health Organisation and numerous national health policies emphasising its role in ensuring a dignified and person-centered end-of-life experience [3–6]. Various metrics have been developed to evaluate the quality of EOL care, including the place of death, timely enrolment in hospice programs, and composite measures of medical care intensity, often referred to as aggressive-care indicators [7–10]. These indicators have been widely applied in research and policy evaluation, yet most were initially developed in oncology populations and may not fully capture the care needs and trajectories of patients with non-cancer terminal illnesses.

For patients with advanced non-cancer illnesses, such as end-stage organ failure or neurodegenerative disease, disease trajectories are often unpredictable and characterised by fluctuating periods of decline [11–15]. This prognostic uncertainty can delay goals-of-care discussions and reduce the likelihood of timely hospice referral [11,16,17]. Even when hospice eligibility criteria are broadened to include non-cancer diagnoses, utilisation rates remain disproportionately low in many healthcare systems [18–20]. In Taiwan, despite the 2009 policy expansion to cover conditions such as dementia, severe brain injury, and end-stage heart, lung, liver, and kidney diseases, national data indicate that hospice enrolment among non-cancer decedents remains below one-quarter [21]. Similar disparities have been observed internationally, where non-cancer patients often receive less specialised palliative input and initiate such care later in the disease course compared with cancer patients [22]. As a result, they are more likely to experience high-intensity interventions, such as repeated emergency department visits, ICU admissions, mechanical ventilation, or cardiopulmonary resuscitation, in the final days of life [23–25].

Most existing research on EOL care for non-cancer patients has either focused on single disease entities, such as dementia, heart failure, or chronic lung disease, or has compared cancer and non-cancer populations without examining variation across non-cancer diagnoses [24,26–28]. These studies have revealed substantial heterogeneity in hospice utilisation patterns, timing of referral, and intensity of medical interventions, suggesting that disease-specific factors may shape EOL care trajectories. However, few large-scale investigations have systematically compared multiple non-cancer conditions within the same healthcare setting to identify barriers and diagnosis-specific needs [28]. In Asian contexts, including Taiwan, there is a particular scarcity of empirical evidence addressing cross-diagnosis differences in hospice enrolment and aggressive care intensity among non-cancer decedents, despite policy frameworks that nominally ensure equal eligibility [18,21].

To address these gaps, we examined a decade-long cohort of hospitalised decedents with hospice-eligible non-cancer diagnoses within a single tertiary medical centre in Taiwan. We investigated hospice utilisation and timing of enrolment across different diagnostic groups, examined variations in place of death and selected aggressive end-of-life care indicators according to hospice care status, and explored diagnosis-specific differences and associated factors related to high aggressive-care scores in the last 28 days of life. Understanding these patterns is essential for developing diagnosis-informed referral strategies and ensuring equitable access to hospice services [29]. Moreover, evidence suggests that early integration of palliative care for non-cancer patients can be both clinically beneficial and cost-effective, reinforcing the importance of optimising resource allocation in this population [30].

## Method

### Design, setting, and participants

This retrospective observational study was conducted at a single tertiary referral hospital in Taiwan. The study protocol was reviewed and approved by the hospital’s Institutional Review Board (IRB No. CE20362A#1; approval date: 1 December 2022), with a waiver of informed consent granted because de-identified data were used. This study was conducted in accordance with the principles of the Declaration of Helsinki.

We included hospitalised adults (aged ≥20 years) who either died during hospitalisation or were discharged in a moribund condition between 1 January 2010 and 31 December 2019. Patients discharged in a moribund condition were defined as those discharged under the impending death discharge policy, indicating that death was clinically expected within a very short time frame. Post-discharge status was confirmed through nurse-initiated telephone follow-up; two patients for whom post-discharge contact with family members could not be established were excluded from the analysis.

**Figure 1.**
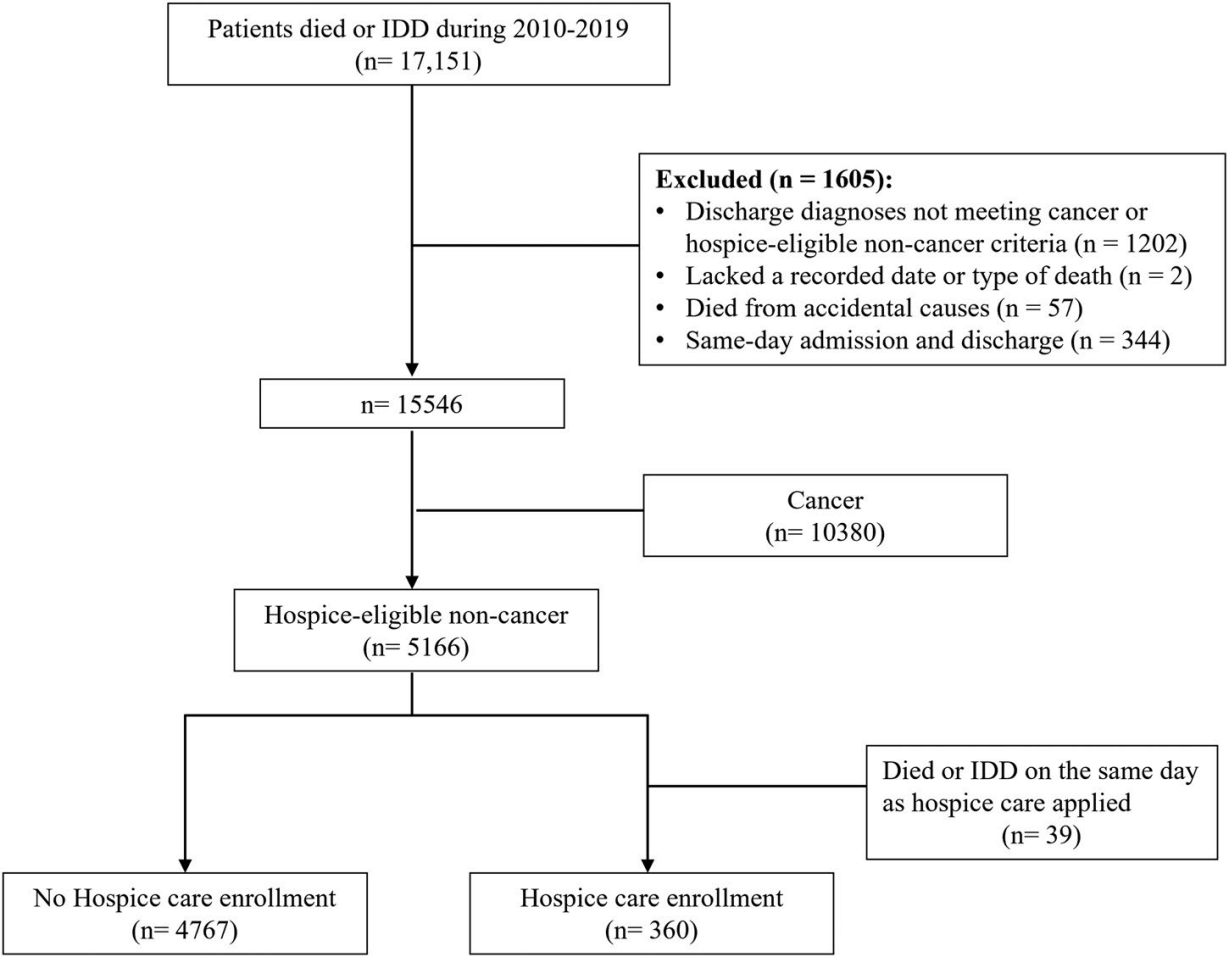
Flowchart of patient selection for the study. Abbreviations: IDD: impending death discharge.

Patients were also excluded if they had diagnoses not meeting hospice-eligible non-cancer criteria, died from accidental causes, had same-day admission and discharge, or had a primary cancer diagnosis. Hospice-eligible non-cancer conditions, defined by National Health Insurance criteria, included end-stage dementia, severe brain injury, advanced heart disease, chronic lung disease, liver failure, and end-stage renal disease (ICD codes listed in Supplementary Table 1). The final cohort was divided into those receiving hospice care and those not receiving hospice care; patients who initiated hospice care and died on the same day were excluded. The selection process is shown in Figure 1.

### Hospice consultation and enrolment process

Patients were initially hospitalised due to acute medical events, symptom exacerbation, or clinical deterioration related to advanced non-cancer chronic illnesses. For patients meeting hospice eligibility criteria, terminal status was determined by two relevant attending specialists in accordance with National Health Insurance regulations. Following eligibility confirmation, the primary attending physician initiated a hospice consultation.

During hospitalisation, hospice care was provided as either hospice shared care in a general ward or hospice inpatient care in a dedicated hospice unit, and some patients transitioned from shared care to inpatient hospice care. After hospice consultation, patients either died during hospitalisation or were discharged under the impending death discharge policy, followed by hospice home care when applicable.

### Study variables and outcomes

Patient characteristics were extracted from the electronic medical record system, including demographic information (age and sex), clinical variables (location of death, terminal diagnosis documentation, year of death, and do-not-resuscitate (DNR) status), as well as health service data such as length of hospital stay, receipt of hospice care, and timing of hospice care enrolment. Terminal diagnosis documentation was defined as a formal determination by two relevant attending specialists that the disease was irreversible and that the patient was expected to die in the near term, as recorded in the medical record.

DNR orders were identified from a dedicated field in the electronic medical record system, which documents both the identity of the signer and the date of consent. In Taiwan, a DNR order indicates a decision to withhold cardiopulmonary resuscitation and related life-sustaining interventions and is generally implemented when a patient is determined to have a terminal, incurable illness with a limited prognosis. When patients retain decision-making capacity, the order is signed by the patient; otherwise, it is signed by a surrogate decision-maker, usually the closest relative, reflecting surrogate decision-making on behalf of the patient. Cardiopulmonary resuscitation (CPR) events and endotracheal intubation were identified based on documented medical orders and execution records. Comorbidity burden was quantified using the modified Charlson Comorbidity Index (CCI), calculated from discharge diagnoses during the index hospitalisation. The ICD-9-CM and ICD-10 coding algorithms described by Quan et al. [31] were applied to identify comorbidities, as summarised in Supplementary Table 2.

The study had two primary outcomes (1): Place of death, categorised as hospital or home, with patients discharged in a moribund condition classified as having died at home; and (2) Aggressive EOL care intensity. Aggressive end-of-life care indicators were defined based on widely used administrative data measures originally proposed by Earle et al. [7,8,32] and later summarised in a systematic review [9,33] and large nationwide cohort studies [10]. Based on data availability and relevance to non-cancer populations, five indicators were selected: hospitalisation for more than 14 days, at least one intensive care unit (ICU) admission, at least one emergency department (ED) visit, CPR, and endotracheal intubation with mechanical ventilation within the last 28 days of life. An Aggressive-care Score was constructed as the sum of these indicators, with higher scores indicating greater care intensity. This composite score was used for descriptive purposes and has not been formally validated as a measurement scale. High-intensity EOL care was defined as an Aggressive-care score ≥4, corresponding to values above the sample median, and was used for analytical stratification rather than as a clinical threshold.

### Statistical analysis

Descriptive statistics were used to summarise patient characteristics, hospice care utilisation, and study outcomes. Continuous variables are presented as medians with interquartile ranges (IQRs) and were compared using the Mann–Whitney *U* test or the Kruskal–Wallis test. Categorical variables are presented as counts and percentages and were compared using the Chi-square test or Fisher’s Exact test. The two primary outcomes were place of death and the Aggressive-care Score (0–5). Univariate comparisons of outcomes were performed according to hospice care status and diagnosis category.

Multivariable logistic regression was performed to identify factors associated with high-intensity EOL care. Covariates included diagnosis group, age, sex, modified Charlson Comorbidity Index, and receipt of hospice care. Odds ratios (ORs) with 95% confidence intervals (CIs) were reported. All statistical analyses were conducted using IBM SPSS Statistics version 22.0 (IBM Corp., Armonk, NY, USA). A two-sided *p*-value <0.05 was considered statistically significant.

## Results

### Baseline characteristics

A total of 5,127 non-cancer decedents were included in the analysis, comprising 110 (2.1%) with dementia, 822 (16.0%) with severe brain injury, 752 (14.7%) with advanced heart disease, 589 (11.5%) with chronic lung disease, 738 (14.4%) with liver failure, 1,353 (26.4%) with end-stage renal disease, and 763 (14.9%) with other conditions. The median age of the study cohort was 76.9 years (IQR, 63.4–85.3), with dementia patients being the oldest (median, 86.4 years) and those with liver failure the youngest (median, 64.5 years; *p* < 0.001). Overall, 64.7% of patients were male, with significant variation across diagnostic groups (*p* < 0.001), ranging from 56.9% in advanced heart disease to 77.0% in chronic lung disease.

Terminal diagnosis documentation was present in 13% of patients, most frequently among those with liver failure (19.1%; *p* < 0.001). The median length of hospital stay was 12 days (IQR, 4–25), with the longest stays observed in liver failure (median, 15 days) and the shortest in dementia and severe brain injury (median, 9 days; *p* < 0.001). Overall, 61.6% of patients had a documented do-not-resuscitate (DNR) order, most often signed by a family member (89.3%). In-hospital deaths accounted for 51.5% of the cohort, with a higher proportion among patients with dementia (68.8%) compared with severe brain injury (49.5%; *p* < 0.001). Patient characteristics are summarised in Table 1; additional comparisons by hospice care status are provided in Supplementary Table 3.

**Table 1.** Baseline demographic and clinical characteristics of hospitalised non-cancer decedents by diagnosis group.

	Total (*n* = 5127)	Non-cancer diagnosis group							*p* value
		Dementia (*n* = 110)	Severe brain injury (*n* = 822)	Advanced heart disease (*n* = 752)	Chronic lung disease (*n* = 589)	Liver failure (*n* = 738)	End-stage renal disease (*n* = 1353)	Others (*n* = 763)	
Age	76.9 (63.4–85.3)	86.4 (82.6–89.5)	73.2 (58.3–83)	79.7 (70–86.9)	83.4 (75.1–88.5)	64.5 (54.3–76.8)	78.0 (65.0–85.9)	77.0 (64.5–84.5)	<0.001**
Sex									<0.001**
Female	1808 (35.3%)	34 (31.3%)	330 (40.2%)	324 (43.1%)	135 (23%)	244 (33.0%)	486 (35.9%)	252 (33%)	
Male	3319 (64.7%)	76 (68.8%)	492 (59.8%)	428 (56.9%)	454 (77%)	494 (67.0%)	867 (64.1%)	511 (67%)	
Terminal diagnosis documentation	668 (13.0%)	12 (10.7%)	147 (17.9%)	80 (10.7%)	67 (11.3%)	141 (19.1%)	195 (14.4%)	49 (6.4%)	<0.001**
Length of hospital stay	12 (4–25)	9 (4–19)	9 (4–22)	11 (5–23)	12 (5–26)	15 (6–27)	13 (4–26)	11 (4–25)	<0.001**
Modified Charlson Comorbidity Index (CCI)									<0.001**
CCI 0–1	2979 (58.1%)	65 (58.9%)	669 (81.3%)	292 (38.9%)	321 (54.5%)	241 (32.6%)	799 (59.1%)	584 (76.5%)	
CCI 2–3	1237 (24.1%)	40 (36.6%)	83 (10.1%)	249 (33.1%)	209 (35.5%)	249 (33.8%)	292 (21.6%)	124 (16.3%)	
CCI ≥ 4	911 (17.8%)	5 (4.5%)	70 (8.5%)	211 (28.0%)	59 (10.0%)	248 (33.6%)	262 (19.4%)	55 (7.2%)	
Place of death									<0.001**
Home	2487 (48.5%)	34 (31.3%)	415 (50.5%)	361 (48.0%)	246 (41.8%)	367 (49.7%)	685 (50.6%)	381 (49.9%)	
Hospital	2640 (51.5%)	76 (68.8%)	407 (49.5%)	391 (52.0%)	343 (58.2%)	371 (50.3%)	668 (49.4%)	382 (50.1%)	
With do-not-resuscitate (DNR)	3156 (61.6%)	59 (53.6%)	578 (70.3%)	389 (51.7%)	335 (56.9%)	521 (70.6%)	845 (62.5%)	438 (57.4%)	<0.001**
Type of DNR									<0.001**
Self-signature	339 (10.7%)	4 (6.7%)	38 (6.5%)	36 (9.2%)	62 (18.5%)	62 (11.9%)	86 (10.2%)	51 (11.6%)	
Relatives signature	2817 (89.3%)	55 (93.3%)	540 (93.5%)	353 (90.8%)	273 (81.5%)	459 (88.1%)	759 (89.8%)	387 (88.4%)	

Abbreviations: CCI: Charlson Comorbidity Index; DNR: do-not-resuscitate.

Values are presented as median (interquartile range) for continuous variables and n (%) for categorical variables.

Differences across groups were tested using the Kruskal–Wallis test for continuous variables and the Chi-square test or Fisher–Freeman–Halton exact test for categorical variables. **p < 0.01.

### Hospice care utilization and timing

Among the 5,127 non-cancer decedents, 360 patients (7.0%) received hospice care, with significant variation across diagnostic groups (*p* = 0.007), with relatively higher enrolment among patients with dementia (10.9%) and lower enrolment among those with advanced heart diseases (5.5%) and others (4.7%). The median interval from hospice enrolment to death was 5 days (IQR, 2–14), with no statistically significant difference among diagnostic groups (*p* = 0.174). Late enrolment was common, with 60.2% of hospice patients admitted within 7 days before death. The proportion of late enrolment did not differ significantly across diagnostic groups (*p* = 0.588). In contrast, early enrolment (>28 days before death) occurred in only 14.4% of hospice patients overall but was more frequent among those with dementia (41.7%) compared with other groups (*p* = 0.009). Hospice care utilisation by diagnosis is presented in Table 2.

**Table 2.** Hospice care utilisation and timing by non-cancer diagnosis group.

	Total (*n* = 5127)	Non-cancer diagnosis group							*p* value
		Dementia (*n* = 110)	Severe brain injury (*n* = 822)	Advanced heart disease (*n* = 752)	Chronic lung disease (*n* = 589)	Liver failure (*n* = 738)	End-stage renal disease (*n* = 1353)	Others (*n* = 763)	
Receiving hospice care	360 (7.0%)	12 (10.9%)	67 (8.2%)	41 (5.5%)	37 (6.3%)	65 (8.8%)	102 (7.5%)	36 (4.7%)	0.007**
Days from hospice enrollment to death	5 (2–14)	5.5 (1–69.3)	3.5 (1–8.8)	6 (1–21)	6 (1–13)	4 (2–15.5)	6 (2–18)	5 (2–18.8)	0.174
Enrolled ≤7 days before death	216 (60.2%)	7 (58.3%)	47 (70.1%)	23 (56.0%)	22 (59.5%)	39 (60.5%)	56 (55.1%)	22 (62.5%)	0.588
Enrolled >28 days before death	52 (14.4%)	5 (41.7%)	4 (6.0%)	9 (22.0%)	5 (13.5%)	6 (9.2%)	17 (16.8%)	6 (17.5%)	0.009**

Values are presented as median (IQR) for continuous variables and *n* (%) for categorical variables.

Differences across groups were tested using the Kruskal–Wallis test for continuous variables and the Chi-square test or Fisher–Freeman–Halton exact test for categorical variables. **p < 0.01.

### Place of death and aggressive medical care by hospice care status

Place of death and aggressive medical care in the last 28 days of life differed substantially between patients who did and did not receive hospice care (*p* < 0.001 for all comparisons). Hospice recipients were more frequently observed to die in hospital (69.7% vs. 50.1%) compared with non-hospice patients.

Hospice care was accompanied by markedly lower use of high-intensity interventions in the last 28 days of life, including emergency department visits (62.2% vs. 75.1%), intensive care unit admissions (39.7% vs. 63.7%), cardiopulmonary resuscitation (1.7% vs. 10.1%), and endotracheal intubation with mechanical ventilation (9.2% vs. 23.2%) (all *p* < 0.001). The median aggressive-care score was 2 (IQR, 1–2) in the hospice group versus 3 (IQR, 2–3) in the non-hospice group (*p* < 0.001). These findings are summarised in Table 3. When stratified by diagnosis, the median aggressive-care score ranged from 2 (IQR, 1–3) in dementia and chronic lung disease to 3 (IQR, 2–3) in most other groups (*p* < 0.001; Supplementary Table 4).

**Table 3.** Place of death and aggressive-care indicators in the last 28 days of life by hospice care status.

	Total (*n* = 5127)	Hospice care status		*p* value
		Receiving (*n* = 360)	Non-receiving (*n* = 4767)	
(A) Place of death				<0.001**
Home	2489 (48.5%)	109 (30.3%)	2380 (49.9%)	
Hospital	2638 (51.5%)	251 (69.7%)	2387 (50.1%)	
(B) Aggressive-care indicators				
>14 days of hospitalization	181 (3.5%)	17 (4.7%)	164 (3.4%)	0.204
≥1 emergency department visit	3803 (74.2%)	224 (62.2%)	3579 (75.1%)	<0.001**
≥1 intensive care unit (ICU) admission	3180 (62%)	143 (39.7%)	3037 (63.7%)	<0.001**
Cardiopulmonary resuscitation (CPR)	489 (9.5%)	6 (1.7%)	483 (10.1%)	<0.001**
Insertion of endotracheal tube with mechanical ventilation	1141 (22.3%)	33 (9.2%)	1108 (23.2%)	<0.001**
Aggressive-care score	3 (2–3)	2 (1–2)	3 (2–3)	<0.001**

Abbreviations: ICU: intensive care unit; CPR: cardiopulmonary resuscitation.

Values are presented as median (IQR) for continuous variables and *n* (%) for categorical variables.

Differences between groups were tested using the Mann–Whitney *U* test for continuous variables and the Chi-square test or Fisher’s exact test for categorical variables. **p < 0.01.

### Factors associated with high aggressive-care score

In the multivariable logistic regression model, advanced heart disease (adjusted OR [aOR], 1.95; 95% CI, 1.18–3.20) and end-stage renal disease (aOR, 1.96; 95% CI, 1.20–3.18) were significantly associated with higher odds of having a high aggressive-care score (≥4) compared with dementia. Older age was associated with lower odds of high aggressive-care scores in a graded manner, with patients aged 65–85 years (aOR, 0.77; 95% CI, 0.67–0.89) and those aged ≥85 years (aOR, 0.43; 95% CI, 0.36–0.51) showing progressively reduced odds compared with patients aged <65 years. Receipt of hospice care was associated with a substantially lower likelihood of high aggressive-care scores (aOR, 0.46; 95% CI, 0.36–0.60; *p* < 0.001). Sex and comorbidity burden (CCI) were not significantly associated with aggressive-care intensity after adjustment. These findings are summarised in Table 4.

**Table 4.** Factors associated with high aggressive-care scores (≥4) in the last 28 days of life by diagnosis group.

	Simple model			Multiple model		
	OR	(95% CI)	*p* value	OR	(95% CI)	*p* value
Diagnosis group						
Dementia	1.00			1.00		
Severe brain injury	1.57	(0.96–2.57)	0.069	1.09	(0.66–1.80)	0.731
Advanced heart disease	2.36	(1.44–3.84)	0.001**	1.95	(1.18–3.20)	0.009**
Chronic lung disease	1.53	(0.93–2.53)	0.095	1.32	(0.79–2.19)	0.286
Liver failure	2.41	(1.48–3.94)	<0.001**	1.65	(1.00–2.73)	0.052
End-stage renal disease	2.52	(1.56–4.07)	<0.001**	1.96	(1.20–3.18)	0.007**
Others	2.35	(1.44–3.83)	0.001**	1.69	(1.03–2.79)	0.038*
Age						
<65	1.00			1.00		
65–85	0.79	(0.69–0.90)	<0.001**	0.77	(0.67–0.89)	<0.001**
≥85	0.43	(0.37–0.51)	<0.001**	0.43	(0.36–0.51)	<0.001**
Male	1.00	(0.89–1.13)	0.977	1.03	(0.91–1.16)	0.646
Modified Charlson Comorbidity Index (CCI)						
CCI 0–1	1.00			1.00		
CCI 2–3	0.86	(0.75–0.99)	0.036*	0.87	(0.75–1.01)	0.064
CCI ≥4	0.96	(0.83–1.13)	0.653	0.91	(0.77–1.07)	0.245
Receiving hospice Care	0.45	(0.35–0.58)	<0.001**	0.46	(0.36–0.60)	<0.001**

Abbreviations: OR: odds ratio; CI: confidence interval; CCI: Charlson Comorbidity Index.

Logistic regression models were used to estimate associations. The simple model included each variable individually, and the multiple model included all variables in the table. *p < 0.05, **p < 0.01.

## Discussion

In this 10-year hospital-based cohort of 5,127 hospitalised decedents with hospice-eligible non-cancer diagnoses, overall hospice utilisation was low (7.0%) and varied substantially across diagnostic groups, with relatively higher utilisation observed among patients with dementia and liver failure, and lower utilisation among those with advanced heart disease and other diagnoses. Most referrals occurred within 7 days before death, indicating that hospice involvement was commonly initiated late in the disease course. Hospice enrolment was associated with lower exposure to high-intensity end-of-life interventions, including emergency department visits, ICU admissions, cardiopulmonary resuscitation, and mechanical ventilation, and with lower aggressive-care scores overall. In multivariable analysis, advanced heart disease and end-stage renal disease were associated with greater odds of high-intensity care, while hospice enrolment was associated with lower odds of lower care intensity. To our knowledge, this is among the first studies to compare aggressive-care scores across multiple non-cancer diagnoses, offering a cross-diagnosis perspective rarely addressed in the literature.

Our findings are consistent with international studies showing persistent disparities in hospice care access and timing for patients with non-cancer terminal illnesses. A U.S. Medicare analysis reported a tendency towards higher use of intensive end-of-life interventions, including ICU admission, among non-cancer decedents, particularly those with advanced cardiopulmonary disease [34]. A systematic review further confirmed that palliative care duration before death is generally shorter for non-cancer patients across settings [35]. Compared with the Japanese national claims study by Togashi et al. [23], the diagnostic distribution of hospitalised non-cancer decedents in our cohort differed substantially. In our study, end-stage renal disease and severe brain injury constituted a relatively larger proportion of hospice-eligible hospitalised decedents, whereas the Japanese study reported that heart disease and respiratory disease accounted for the largest proportions of inpatient deaths; notably, dementia accounted for a relatively small proportion of decedents in both cohorts.

In our cohort, hospice enrolment varied across diagnoses. Patients with dementia showed a relatively higher hospice enrolment rate and a higher proportion of early enrolment, possibly reflecting earlier recognition of progressive decline and caregiver needs in selected hospitalised cases. This may be partly explained by the more predictable trajectory of decline in dementia [36] and differing palliative care needs [37] across disease groups. These findings are consistent with prior studies demonstrating variation in end-of-life care patterns across patient groups with different underlying conditions [38]. However, compared with population-based studies, the proportion of patients with dementia in our cohort was substantially lower [39], likely reflecting underrepresentation of individuals who died without hospitalisation, particularly those residing in long-term care facilities or receiving home-based care. Accordingly, findings related to hospice enrolment and end-of-life care for dementia may primarily reflect patterns among hospitalised patients. In contrast, patients with severe brain injury showed predominantly late referrals, with most enrolments occurring within the final week of life, a pattern that may reflect referral decisions made in response to acute clinical deterioration rather than proactive care planning. Notably, a subset of patients who died during the index hospitalisation were not formally documented as having a terminal diagnosis, highlighting the challenge of timely prognostic recognition in non-cancer conditions and potentially contributing to delayed hospice referral and continued high-intensity care near the end of life. By examining diagnosis-specific patterns within a uniform healthcare system, our study extends these observations and highlights the need for targeted strategies to improve early palliative integration for non-cancer populations.

These findings carry important implications for both clinical practice and health policy. Clinically, the marked variation in hospice utilisation across diagnoses suggests that referral decisions are influenced not only by patient prognosis but also by disease-specific care cultures and provider experience. Diagnosis-informed referral triggers may facilitate timely palliative integration, particularly for conditions with unpredictable trajectories such as heart failure or dementia [11,40]. From a policy perspective, addressing structural inequities in hospice access is essential. Evidence suggests that diagnosis-based eligibility rules and uneven service distribution may disproportionately disadvantage non-cancer patients [29]. Early palliative care integration has been associated with improved patient and caregiver outcomes and with reduced end-of-life healthcare costs [30,39,41]. Beyond policy initiatives, education of healthcare providers, patients, and families is also critical to support earlier, quality-of-life–focused discussions and timely palliative referral in non-cancer populations. Aligning reimbursement models and care pathways to support earlier, needs-based palliative referrals may therefore improve both quality and efficiency of end-of-life care.

Beyond enrolment patterns, place of death and intensity of medical interventions also varied substantially, reflecting cultural preferences and healthcare system structures. Within this hospitalised cohort, hospice recipients were more likely to die in hospital than non-recipients (69.7% vs. 50.1%), which may reflect the availability of inpatient hospice beds, patient or family preferences, or challenges in arranging home-based end-of-life care. Although dying at home is recognised internationally as one of the quality indicators for end-of-life care [42], in Taiwan, religious beliefs and the structure of the National Health Insurance system are associated with a preference for hospital-based death. However, in this hospital-based cohort, observed place-of-death patterns primarily reflect trajectories following a terminal hospitalisation and may not fully capture end-of-life care completed entirely in community or home settings.

In a hospital-based study from Taiwan, Ko et al. reported that among non-cancer decedents, ICU admission within 30 days of death occurred in ∼47–58%, death in the ICU in 34–45%, and cardiopulmonary resuscitation shortly before death in 5–13%, depending on the study period [25]. In comparison, our cohort of hospitalised non-cancer decedents demonstrated comparably high levels of aggressive end-of-life care, particularly for ICU utilisation and in-hospital death, while cardiopulmonary resuscitation rates were comparable. Diagnosis-specific variation in aggressive-care scores suggests that disease trajectories and specialty care models are associated with different end-of-life treatment patterns. In our cohort, advanced heart disease and end-stage renal disease were associated with higher odds of high-intensity care, consistent with prior reports describing escalated interventions during episodes of acute decompensation [14,15]. Hospice enrolment was linked to lower observed rates of emergency department visits, ICU admissions, cardiopulmonary resuscitation, and mechanical ventilation, but not prolonged hospitalisation. This lack of difference may be due to discharge barriers, such as insufficient family caregiving capacity at home, cultural preferences for dying in hospital, or limited access to home-based hospice services, or to established care plans in which patients and families elect to remain in inpatient hospice until death. Similar patterns have been observed in disease-specific cohorts, where palliative care reduced ICU use, intubation, and CPR in patients with kidney failure on maintenance dialysis [43] and end-stage heart failure [44]. Systematic reviews further support that targeted palliative interventions can reduce aggressive end-of-life care without compromising patient outcomes [33].

This study has several limitations. First, it was conducted at a single tertiary medical centre in Taiwan, where inpatient hospice resources and cultural preferences for hospital-based death may differ from other settings, limiting generalisability. Second, the retrospective design lacked key clinical variables—such as functional status, symptom burden, and care preferences—that may influence referral timing and care intensity [45]. In addition, focusing on the last 28 days of life may not capture earlier palliative care needs, treatment limitations, or hospice interventions, particularly in chronic non-cancer conditions. Information on goals of care, advance care planning, and the clinical rationale underlying treatment decisions was not consistently documented and could not be systematically analysed. Third, the aggressive-care indicators used here, while adapted from commonly used measures, have not been formally validated as a composite score and may not fully capture care quality, particularly in non-cancer populations. Given the retrospective observational design, causal relationships between hospice enrolment and end-of-life care outcomes cannot be established. Hospice referral and outcomes may be influenced by unmeasured factors, such as symptom severity, functional status, or patient and family preferences, introducing potential selection and survival biases. Fourth, our analysis was confined to the index hospitalisation, without accounting for outpatient or home hospice care that might affect end-of-life hospitalisation patterns; consequently, comorbidity burden may be underestimated, as conditions managed during prior admissions or at other institutions were not fully captured. Future research should integrate multicentre data, incorporate patient-reported outcomes, and evaluate tailored referral triggers to optimise timing and content of palliative care for diverse non-cancer diagnoses.

## Conclusion

In this 10-year cohort of hospitalised non-cancer decedents, hospice care was used by only 7.0% of patients, most often in the final week of life, with substantial variation across diagnoses. Hospice enrolment was associated with lower aggressive-care scores, primarily due to fewer emergency visits, ICU admissions, cardiopulmonary resuscitations, and mechanical ventilations, yet diagnosis-specific differences persisted. By providing a cross-diagnosis comparison of aggressive-care scores within a single healthcare system, these findings underscore opportunities to refine diagnosis-informed referral approaches and policy measures aimed at facilitating more timely palliative care integration.

## Supplementary Material

Supplementary Table 3.docx

Supplementary Table 1.docx

Supplementary Table 4.docx

Supplementary Table 2.docx

## Data Availability

The data that support the findings of this study are not publicly available due to institutional data protection policies, but are available from the corresponding author upon reasonable request and with permission from Taichung Veterans General Hospital, in accordance with institutional regulations.
