# Supplementary material for: Disparities in hospice enrollment timing and end-of-life care intensity across non-cancer diagnoses: a 10-year hospital-based cohort study
Source: Ann Med. 2026 May 16;58(1):2670058. doi: 10.1080/07853890.2026.2670058 (PMC13182166; doi:10.1080/07853890.2026.2670058)
Supplement: Supplementary Table 3.docx [file IANN_A_2670058_SM6993.docx]

| Supplementary Table 3. Baseline characteristics of hospitalized non-cancer decedents by hospice care status | | | | |
| --- | --- | --- | --- | --- |
|  | Total (n=5127) | Hospice Care Status | | *p* value |
|  |  | Receiving (n=360) | Non-receiving (n=4767) |  |
| Age | 76.9 (63.4 - 85.3) | 76 (64.1 - 86.4) | 77 (63.3 - 85.3) | 0.615 |
| Sex |  |  |  | 0.823 |
| Female | 1808 (35.3%) | 125 (34.7%) | 1683 (35.3%) |  |
| Male | 3319 (64.7%) | 235 (65.3%) | 3084 (64.7%) |  |
| Terminal diagnosis documentation | 668 (13.0%) | 238 (66.1%) | 430 (9.0%) | <0.001** |
| Length of hospital stay | 12 (4 - 25) | 20 (10 - 36) | 11 (4 - 24) | <0.001** |
| Diagnosis |  |  |  | 0.007** |
| Severe brain injury | 822 (16.0%) | 67 (18.6%) | 755 (15.8%) |  |
| End-stage renal disease | 1353 (26.4%) | 102 (28.3%) | 1251 (26.2%) |  |
| Advanced heart disease | 752 (14.7%) | 41 (11.4%) | 711 (14.9%) |  |
| Liver failure | 738 (14.4%) | 65 (18.1%) | 673 (14.1%) |  |
| Chronic lung disease | 589 (11.5%) | 37 (10.3%) | 552 (11.6%) |  |
| Dementia | 110 (2.1%) | 12 (3.3%) | 98 (2.1%) |  |
| Others | 763 (14.9%) | 36 (10.0%) | 727 (15.3%) |  |
| Modified Charlson Comorbidity Index (CCI) | |  |  | <0.001** |
| CCI 0-1 | 2979 (58.1%) | 156 (43.3%) | 2823 (59.2%) |  |
| CCI 2-3 | 1237 (24.1%) | 94 (26.1%) | 1143 (24%) |  |
| CCI ≥ 4 | 911 (17.8%) | 110 (30.6%) | 801 (16.8%) |  |
| Year of death |  |  |  | <0.001** |
| 2010 | 505 (9.8%) | 3 (0.8%) | 502 (10.5%) |  |
| 2011 | 551 (10.7%) | 5 (1.4%) | 546 (11.5%) |  |
| 2012 | 557 (10.9%) | 4 (1.1%) | 553 (11.6%) |  |
| 2013 | 524 (10.2%) | 14 (3.9%) | 510 (10.7%) |  |
| 2014 | 505 (9.8%) | 19 (5.3%) | 486 (10.2%) |  |
| 2015 | 549 (10.7%) | 40 (11.1%) | 509 (10.7%) |  |
| 2016 | 507 (9.9%) | 58 (16.1%) | 449 (9.4%) |  |
| 2017 | 488 (9.5%) | 73 (20.3%) | 415 (8.7%) |  |
| 2018 | 469 (9.1%) | 76 (21.1%) | 393 (8.2%) |  |
| 2019 | 472 (9.2%) | 68 (18.9%) | 404 (8.5%) |  |
| With do-not-resuscitate (DNR) | 3156 (61.6%) | 237 (65.8%) | 2919 (61.2%) | 0.084 |
| Type of DNR |  |  |  | 0.322 |
| Self-signature | 339 (10.7%) | 30 (12.7%) | 309 (10.6%) |  |
| Relatives signature | 2817 (89.3%) | 207 (87.3%) | 2610 (89.4%) |  |
| Abbreviations: CCI, Charlson Comorbidity Index; DNR, do-not-resuscitate.  Values are presented as median (interquartile range) for continuous variables and n (%) for categorical variables.  Differences between groups were tested using the Mann–Whitney U test for continuous variables and the Chi-square test or Fisher’s exact test for categorical variables. *p<0.05, **p<0.01. | | | | |
