# Supplementary material for: Disparities in hospice enrollment timing and end-of-life care intensity across non-cancer diagnoses: a 10-year hospital-based cohort study
Source: Ann Med. 2026 May 16;58(1):2670058. doi: 10.1080/07853890.2026.2670058 (PMC13182166; doi:10.1080/07853890.2026.2670058)
Supplement: Supplementary Table 1.docx [file IANN_A_2670058_SM6992.docx]

| Supplementary Table 1. List of ICD-9 and ICD-10 diagnostic codes for hospice care in Taiwan | | |
| --- | --- | --- |
| Diagnostic cluster | ICD-9 | ICD-10 |
| Dementia | 290.x, 291.1, 291.2, 292.82, 294.1, 294.8, 294.9, 331.0, 331.1, 331.2 | F01.x-F03.x, F10.27, F10.97, F13.27, F13.97, F18.27, F18.97, F19.27, F19.97, G30.x, G31.x |
| Severe brain injury  (Stroke and others) | 430.x–438.x | G45.x, G46.x, H34.0, I60.x–I69.x |
| Advanced Heart disease | 398.90, 398.91, 402.01, 402.11, 402.91, 404.01, 404.03, 404.11, 404.13, 404.91, 404.93, 414.8, 425.2, 425.4, 425.5, 425.7, 425.8, 425.9, 428.x, 779.8 | I09.81, I09.9, I11.0, I13.0, I13.2, I25.5, I42.0, I42.5–I42.9, I43.x, I50.x, P29.0 |
| Chronic Lung disease | 415.19, 416.8, 416.9, 490.x–505.x, 506.4, 508.1, 508.8, 519.1  277.00 | I27.8x, I27.9, J40.x–J47.x, J60.x–J67.x, J68.4, J70.1, J70.2, J70.3  E84.0 |
| Liver failure | 070.22, 070.23, 070.32, 070.33, 070.44, 070.54, 070.59, 456.0, 456.1, 456.8, 570, 571.x-573.x | B18.x, I85.0x, I86.4, K70.x, K71.0, K71.1x, K71.3, K71.4, K71.5x, K71.6, K71.7, K71.8, K71.9, K72.1x, K72.9x, K73.x, K74.x, K75.x, K76.x, K77 |
| End-stage renal disease | 583.6, 583.7, 584.5, 584.6, 584.7, 584.8, 584.9, 585, 586, 593.9 | N17.x, N18.4, N18.5, N18.6, N18.9, N19, N18.0, N18.8 |
| Others  (Motor neuron diseases, Myelodysplastic syndromes, Age-related frailty, advanced neurodegenerative diseases, and other specified tremor disorders, Rare diseases) | 340, 332.x, 333.0, 333.1  335.20, 335.21, 335.22, 335.24, 335.29  284.9, 285, 238.7  797  277.00, 331.7, 333, 333.4, 334.x, 335.10, 335.20, 335.21, 335.9, 349.89, 356.0, 359.0, 359.1, 416.0, 719.60, 719.61, 719.62, 719.63, 719.64, 723.5, 754.89, 757.39, 780.03, 781.3, 781.91, 781.99  237.72, 251.1, 253.2, 253.3, 255.1, 255.4, 255.5, 258.1, 259.4, 259.8, 266.2, 269.3, 270.0-270.8, 271.0, 271.1, 271.4, 271.8, 272.0, 272.3, 272.6-272.9, 275.1, 275.3, 275.49, 277.00, 277.1-277.3, 277.5, 277.6, 277.8, 277.9, 279.04, 279.05, 279.11, 279.12, 279.2, 279.3, 279.8, 282.4, 283.11, 283.2, 287.1, 287.8, 288.1, 289.8, 299.8x, 319, 330.0, 330.1, 330.8, 331.89, 333.0, 333.4, 333.91, 334.0., 334.1, 334.3, 334.8, 335.10, 335.20-335.22, 335.24, 335.29, 335.9, 340, 341.8, 345.11, 345.2, 345.3, 356.0-356.2, 359.2, 359.8, 362.70, 416.0, 448.0, 576.2, 628.1, 705.0, 728.11, 731.0, 742.2, 743.41, 743.44, 747.89, 751.5, 751.60, 751.69, 753.14, 755.38, 755.39, 755.53-755.59, 755.67, 755.69, 755.8, 756.0, 756.3, 756.4, 756.51, 756.52, 756.54, 756.54, 756.56, 756.59, 756.8x, 756.9, 757.1, 757.2, 757.31, 757.33, 757.39, 758.3, 758.8x, 759.1, 759.5-759.7, 759.81, 759.89, 775.1, 780.51, 780.53, 780.57, 785.59, 271.3+270.1 | G35, G20, G21.x, G23.x, G10  G12.2x  D46.x  R54  E84.9, G10, G11.x, G12.21, G12.9, G31.2, G32.8x, G60.2, G71.0, G71.2, G90.3, I27.0, Q74.3, Q81.x, R27.x, R29.8xx, R40.3  D56.0, D56.1, D59.3, D59.5, D68.59, D71, D80.5, D81.0, D81.1, D81.2, D81.810, D81.819, D81.9, D82.0, D82.1, D82.4, D84.1, D84.8, E16.1, E20.1, E23.0, E26.81, E27.49, E31.0, E34.3, E34.8, E61.5, E70.0, E70.1, E70.20, E70.21, E70.41, E70.8, E70.9, E71.0, E71.110, E71.118, E71.120, E71.19, E71.3x, E71.41, E71.510, E71.511, E71.520, E71.521, E71.528, E71.529, E71.540, E72.03, E72.04, E72.11, E72.19, E72.20, E72.23, E72.29, E72.3, E72.4, E72.51, E72.52, E72.59, E72.8, E74.01, E74.02, E74.03, E74.04, E74.09, E74.21, E74.4, E74.8, E75.00, E75.11, E75.19, E75.2x, E75.4, E75.5, E76.x, E76.3, E77.0, E77.1, E77.8, E78.0, E78.3, E78.70, E78.71, E78.72, E79.1, E80.2x, E83.01, E83.09, E83.31, E83.32, E83.39, E84.9, E85.1, E88.01, E88.1, E88.40, E88.41, E88.49, E88.9, F78, F84.2, F84.8, G10, G11.1, G11.3, G11.4, G12.2x, G12.9, G23.0, G25.82, G31.82, G31.89, G35, G37.8, G40.311, G47.35, G60.0, G71.0, G71.11, G71.13, G71.2, G71.8, G87.0, H35.50, H49.811, H35.50, H49.81x, I27.0, I78.0, K83.1, L74.4, M61.10, M61.11x, M61.12x, M61.13x, M61.14x, M61.15x, M61.16x, M61.17x, M61.18, M61.19, M88.x, P70.2, Q04.3, Q13.4, Q28.8, Q43.8, Q44.7, Q61.19, Q71.6x, Q72.7x, Q74.0, Q74.8, Q75.1, Q75.4, Q77.2, Q77.3, Q77.4, Q77.8, Q78.0, Q78.1, Q78.2, Q78.3, Q79.6, Q79.8, Q80.2, Q80.3, Q80.4, Q81.9, Q82.3, Q82.4, Q82.8, Q85.02, Q85.1, Q85.8, Q87.0, Q87.1, Q87.2, Q87.3, Q87.89, Q89.1, Q89.7, Q89.8, Q93.5, Q93.88, Q93.89, Q97.8, Q98.8, Q99.8, E74.31+E70.0, E71.120+E72.11 |
