# Supplementary material for: Disparities in hospice enrollment timing and end-of-life care intensity across non-cancer diagnoses: a 10-year hospital-based cohort study
Source: Ann Med. 2026 May 16;58(1):2670058. doi: 10.1080/07853890.2026.2670058 (PMC13182166; doi:10.1080/07853890.2026.2670058)
Supplement: Supplementary Table 4.docx [file IANN_A_2670058_SM6991.docx]

| Supplementary Table 4. Aggressive-care scores in the last 28 days of life by non-cancer diagnosis group | | | | | | | | | |
| --- | --- | --- | --- | --- | --- | --- | --- | --- | --- |
|  | Total (n=5127) | Non-Cancer Diagnosis Group | | | | | | | *p* value |
|  |  | Dementia (n=110) | Severe brain injury (n=822) | Advanced heart disease (n=752) | Chronic lung disease (n=589) | Liver failure (n=738) | End-stage renal disease (n=1353) | Others (n=763) |  |
| Aggressive-Care Score | 3 (2 - 3) | 2 (1 - 3) | 3 (2 - 3) | 3 (2 - 3) | 2 (1 - 3) | 3 (2 - 3) | 3 (2 - 3) | 3 (2 - 3) | <0.001** |
| Values are presented as median (IQR) for continuous variables.  Differences across groups were tested using the Kruskal–Wallis test. *p<0.05, **p<0.01. | | | | | | | | | |
