# Supplementary material for: Disparities in hospice enrollment timing and end-of-life care intensity across non-cancer diagnoses: a 10-year hospital-based cohort study
Source: Ann Med. 2026 May 16;58(1):2670058. doi: 10.1080/07853890.2026.2670058 (PMC13182166; doi:10.1080/07853890.2026.2670058)
Supplement: Supplementary Table 2.docx [file IANN_A_2670058_SM6990.docx]

| Supplementary Table 2. Charlson Comorbidity Index (CCI) Categories with Corresponding ICD Codes | | | | | |
| --- | --- | --- | --- | --- | --- |
| Comorbidity Category | CCI Score | ICD-9-CM Codes | | ICD-10-CM Codes | |
| Heart failure and cardiomyopathy | 1 | 428.x  398.91  402.01  402.11  402.91  404.01  404.03  404.11  404.13  404.91 | 404.93  425.4  425.5  425.6  425.7  425.8  425.9  428 | I11.0  I13.0  I13.2  I25.5  I42.0  I42.5  I42.6 | I42.7  I42.8  I42.9  I43  I50  P29.0 |
| Chronic pulmonary disease | 1 | 490-496  500-505  506.4  508.1  508.8 | | J40-J47  J60-J67  J68.4  J70.1  J70.3 | |
| Diabetes without chronic complication | 1 | 249.0–249.3  249.9  250.0–250.3  250.7–250.9 | | E08.0  E08.1  E08.6  E08.8  E08.9  E09.0  E09.1  E09.6  E09.8  E09.9  E10.0  E10.1  E10.6  E10.8 | E10.9  E11.0  E11.1  E11.6  E11.8  E11.9  E13.0  E13.1  E13.6  E13.8  E13.9 |
| Diabetes with chronic complication | 2 | 250.4  250.5  250.6  250.7 | | E08.2  E08.3  E09.2  E09.3  E10.2 | E10.3  E11.2  E11.3  E13.2  E13.3 |
| Any malignancy, including lymphoma and leukemia, except malignant nonmelanoma neoplasm of skin | 2 | 140–149  150–159  160–169  174–176  179–189  190–195  199.1  200–208  238.6 | | C00-C09  C10-C19  C20-C29  C30-C34  C37-C41  C43  C45-C50 | C51-58  C60-63  C76  C80.1  C81-C85  C88  C90-C99 |
| Dementia | 1 | 290.0  290.1  290.2  290.3  290.4  294.0  294.1 | 294.2  294.8  331.0  331.1  331.2  331.7  797 | F01  F02  F03  F04  F05  F06.1  F06.8  G13.2  G13.8 | G30  G31.0  G31.1  G31.2  G91.4  G94  R41.81  R54 |
| Moderate or Severe Liver Disease | 3 | 456.0  456.1  456.2  572.2  572.3  572.4  572.8 | | I85.0  I86.4  K70.4  K71.1  K72.1  K72.9  K76.5  K76.6  K76.7 | |
| Renal (Mild or Moderate) | 1 | 582.x  583.0–583.7  585.x  586  588  403.00–403.90  404.00–404.91 | | V42.0  I12.9  I13.0  I13.10  N03  N05  N18.1 | N18.2  N18.3  N18.4  N18.9  Z94.0 |
| Renal (Severe) | 3 | 582.x  583.0–583.7  585.x  586  588  403.01–403.91  404.02–404.93 | | V45.11  V45.12  V56.0  V56.1  V56.2  V56.31  V56.32  V56.8 | I12.0  I13.11  I13.2  N18.5  N18.6  N19  N25.0  Z49  Z99.2 |
